# An Assessment Method for Identifying Acceptable and Effective Ways to Present Demands to an Adult With Dementia

**DOI:** 10.1007/s40617-020-00409-y

**Published:** 2020-01-28

**Authors:** Emma E. M. Williams, Rebecca A. Sharp, Carolien Lamers

**Affiliations:** 1 School of Psychology, Bangor University, Bangor, Gwynedd LL57 2DG UK; 2 North Wales Clinical Psychology Programme, Bangor University, Bangor, Wales UK

**Keywords:** Demand assessment, Dementia

## Abstract

Simple instructions are often recommended for presenting demands to people with dementia; however, simple instructions may be perceived as authoritative and may not be appropriate for all individuals. We conducted a demand assessment with a woman with dementia who engaged in problem behaviors in response to direct instructions. We measured latency to compliance and verbal behavior when demands were presented as questions, rules, simple instructions, or demands embedded in social chatter. In contrast to the other conditions, simple instructions resulted in the most undesirable behavior and were least likely to evoke compliance. We conducted an intervention in which demands were phrased as requests for assistance.

There is growing evidence for the use of behavioral interventions in clinical settings for older adults such as dementia settings. Many interventions that could be used in dementia settings stem from research conducted with other populations (i.e., children with autism spectrum disorder and people with intellectual disabilities; Trahan, Kahng, Fisher, & Hausman, 2011). Some of these tactics might require adaptations for older adults to make them socially valid and effective. It is often recommended that people should be given short, simple instructions to facilitate compliance (Robinson, White, & Houchins, 2006). However, for older adults with long learning histories of complex social interactions, being giving short instructions without social niceties might be perceived as rude, or they might lack acceptability for clients, staff members, and family. Tranvåg, Petersen, and Nåden (2014) found that life stories, as well as preserving manners and values, were highly important to people with dementia. From a behavior-analytic perspective, manners and values are learned behaviors, and in an older adult, they are likely to have been reinforced over the person’s lifetime and therefore may be persistent. We could not find any empirical studies explicitly on social values or rules in people with dementia. However, there is some research that shows challenging behavior may be an indicator that the way individuals interact with people with dementia behavior might violate these values for the person with dementia. For example, Ryan, Hummert, and Boich (1995) reviewed different approaches for conversing with older adults and found that when people were spoken to in short sentences, in baby talk, and in patronizing words, challenging behaviors were more likely to be observed.

On occasion, it is necessary to place a demand in settings in which older adults reside, but it is important to do so in a way that is appropriate and acceptable and that results in the person meeting the demand. For example, an adult with dementia in a care home might be asked to move aside to allow staff to pass in the corridor to provide emergency medical care to another resident. Therefore, it is necessary to explore two aspects of the best way to deliver demands to older adults with dementia. First, we need to seek ways of delivering demands that are effective discriminative stimuli for the requested behavior. There is emerging evidence that the behavior of some adults with dementia might be under faulty stimulus control (Buchanan, Houlihan, & Linnerooth, 2010), and therefore discrimination is affected adversely. Christenson, Buchanan, Houlihan, and Wanzek (2011) showed that adults with dementia were more likely to comply with demands when the demands were clear, concise, and direct. There is existing literature on systematically manipulating the type of demand to measure the effect on compliance and disruptive behavior (i.e., Roscoe, Rooker, Pence, Longworth, & Zarcone, 2009); however, there is little on evaluating how demands are placed.

Second, it is important that behavior-analytic methods are adapted to be socially acceptable for use with adults with dementia. Adults with dementia are likely to have learning histories with regard to what constitutes an acceptable or polite social interaction with other adults. For example, Williams, Herman, Gajewski, and Wilson (2008) found that demands from staff to residents in care homes using “elderspeak” (i.e., adopting patronizing comments and high-pitched tones and using oversimplified language) frequently resulted in undesired behaviors. One way to determine social acceptability is to measure client responses to how demands are delivered (i.e., by measuring compliance, as well as other behavior such as verbal behavior). The purpose of our study was to explore the best and most appropriate method for delivering demands to a woman with dementia who engaged in disruptive verbal behavior and was unlikely to comply when demands were presented as simple instructions.

## Method

### Participants and Setting

Mary was 82 years old and had a diagnosis of Alzheimer’s disease (received 6 years prior to the study). She lived in a large, specialized assisted-living home for people with dementia (containing 84 beds). Mary could follow one-step instructions, partook in group activities with some verbal prompting from staff, and spoke in full sentences. However, she was unable to partake in complex conversations involving long sentences. There were some shared living spaces within the residential home, and while in those areas, Mary frequently had to comply with demands to ensure her own and others’ safety. During times when an unavoidable demand was given (such as a requirement for Mary to maneuver in a communal corridor when others needed to pass, or during personal care), she frequently protested, raised her voice, and reprimanded other people. It was becoming increasingly difficult for staff members to approach Mary with demands without occasioning verbal aggression.

### Measurement and Interobserver Agreement

We defined *compliance* as engaging in the behavior specified in the instruction (i.e., picking up a pencil, touching the tip to the paper, and making strokes), and this was recorded during demands presented as questions, chat and instruction, and rules. We defined *rude behavior* as verbal statements that might be perceived as rude by another person, such as name calling, swearing, commenting in a derogatory way on someone’s appearance or personal attributes, and reprimanding another person. We also included nonvocal behaviors such as eye rolling, “tutting” (an audible noise created by pushing the tongue against the lips, teeth, or cheeks, often to show displeasure), and face pulling. Response latency in seconds was measured using a stopwatch from the presentation of the discriminative stimulus to the occurrence of the behavior.

A second observer recorded data in 32% of trials across all phases. Each of the two trained independent observers (graduate students in behavior analysis) recorded the latency to rude behavior and the latency to compliance in each trial. We calculated interobserver agreement (IOA) by dividing the smaller of the two recorded latencies by the larger latency and multiplying by 100 (for each behavior). Mean IOA was 96% for rude behavior (range 93%–99%) and 98% for compliance (range 94%–100%).

We used a trial-based assessment with multiple conditions and a B-A-B withdrawal design for the intervention phase (Tawney & Gast, 1984).

### Procedure

#### Demand Assessment

We assessed which method of presenting the demand would evoke compliance and rude behavior (Table 1). Each trial was composed of one of four conditions, and each condition was repeated five times over a 2-week period during naturally occurring opportunities (20 trials in total). The four conditions were (a) a demand placed as an instruction, in which one sentence was delivered stating the demand; (b) a demand placed as a rule, in which one sentence was stated issuing the demand, followed by a sentence stating a consequence; (c) a demand placed as a question, in which one sentence was delivered asking if Mary would like to carry out the demand; and (d) a demand embedded in chat, in which 10 s of talking about preferred activities was inserted before or after the demand. Trials were 120 s in duration, and we conducted the assessment in Mary’s room. If there was no response after 120 s, the trial was terminated. We conducted the assessment during activities Mary preferred (as reported by staff) to ensure we were assessing how the demand was placed rather than the effect of aversive demands on the target behaviors.

**Table 1:** The Four Conditions Used in the Demand Assessment and an Example of the Phrasing of Each

Condition	Example Phrasing of Demand
Instruction	“Please color the picture.”
Question	“Would you like to color the picture?”
Chat and instruction	10 s of chat before and after “Color the picture.”
Rule	“If you color the picture, we’ll get a cup of tea.”

#### Demands as Mands for Assistance

Based on the results of the assessment, we evaluated the effectiveness of phrasing demands as questions. Specifically, we instructed staff to present demands as mands for assistance with a choice of two specific responses for Mary. For example, “Mary, I really need your help with something. We have to do this [the demand]. Would it be better if we did it this way or that way?” Staff thanked Mary for her help contingent on compliance with the demand and provided no response contingent on rude behavior. If Mary did not respond after 30 s, staff walked away.

#### Demands as Instructions

After six trials of demands as mands for assistance, we instructed staff to present demands as one-step instructions (e.g., “Mary, please sit in that chair.”). After three trials, we returned to a phase in which demands were placed as mands for assistance. We conducted trials during naturally occurring opportunities to place a demand on Mary. No more than two trials were conducted in a day.

## Results

### Demand Assessment

**Figure 1.**
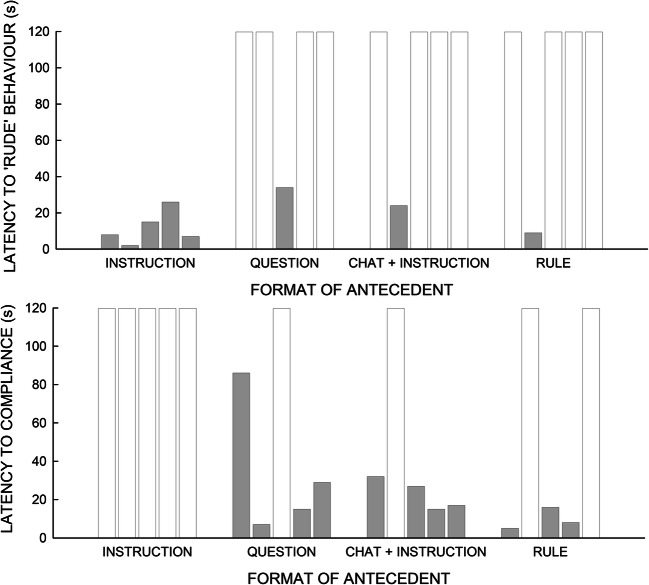
Results of the demand assessment showing latency to rude and compliance behavior during each condition. Trial duration was a maximum of 120 s; therefore, bars that reach 120 s on the graph (white bars) show that rude behavior or compliance did not occur during the trial.

When demands were presented as an instruction, Mary engaged in rude behavior in each trial (latency 2–26 s; Figure 1). Compliance did not occur during any demand phrased as an instruction. When a demand was presented phrased as a question, Mary engaged in rude behavior in one trial (latency 37 s), and she engaged in compliance in four trials (latency 7–86 s). During trials in which demands were embedded in chat, Mary engaged in rude behavior once (latency 23 s). Mary engaged in compliance in four trials (latency 15–38 s). The shortest latency to compliance was during trials in which demands were phrased as rules (5 s), although noncompliance occurred twice during this condition.

### Demands as Mands for Assistance or Instructions

**Figure 2.**
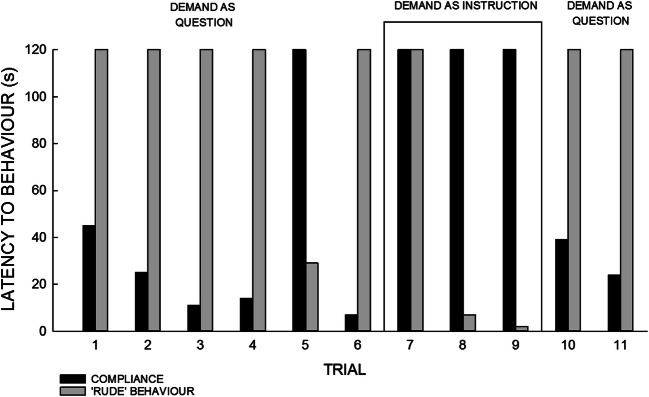
Latency to both compliance and rude behavior during the intervention in which demands were phrased as a question and a mand for help by staff. Trial duration was a maximum of 120 s; therefore, bars that reach 120 s on the graph show that behavior did not occur in that trial.

When staff phrased demands as mands for assistance with a choice of responses, Mary complied and did not engage in rude behavior in five out of six trials (latency 7–45 s; Figure 2). In the fifth trial, she did not comply with the demand and engaged in rude behavior (latency 31 s). However, when demands were placed as instructions, Mary never complied and quickly engaged in rude behavior in two out of three sessions (latency 2 s and 7 s, respectively). When we returned to phrasing demands as mands for assistance, compliance occurred (latency 21–41 s), and Mary did not engage in rude behavior in either trial.

## Discussion

We conducted an assessment to determine which phrasing of demands was most likely to result in compliance and minimize rude behavior in an older adult with dementia. We found that demands phrased as questions were most likely to result in compliance and least likely to result in rude behavior. We subsequently evaluated an intervention in which demands were phrased as mands for assistance with an embedded choice of two responses. We found that the intervention resulted in the participant being more likely to comply with demands and less likely to engage in rude behavior. We used this approach over others for our participant (e.g., instructions embedded in chat), because during casual observations, we noted that Mary could become unresponsive when complex sentences were used to interact with her. Similarly, we predicted that the embedded choice was more likely to be implemented consistently across staff (i.e., it would minimize the risk of different staff members using different amounts or quality of chatting). However, we did not record data on the procedural integrity of staff implementation after the study. Additionally, staff indicated that Mary experienced issues in stimulus control—that she often behaved as though she worked in the home rather than lived in the home as a resident. Therefore, the use of choice aligned with the staff’s beliefs that providing a choice would give Mary perceived control over the environment.

Christenson et al. (2011) found that demands delivered in a clear, concise, and direct manner were more likely to result in compliance. We found that the most important factor affecting our participant’s responding was the phrasing of the demand. For adults with long learning histories of social interactions with other adults, careful consideration as to the nature of social interactions and demands is needed. For example, simple instructions presented tactfully or in a certain way may indeed be acceptable for some older adults. This may account for differences in compliance with different staff members’ requests. There is unlikely to be a one-size-fits-all approach to delivering acceptable demands to people with dementia. Although instructions should be delivered in a way to evoke the desired response to the demand, social niceties should be considered. For example, the care staff placing demands on people with dementia are often younger than their clients, and there are likely social rules for how younger people interact with older people. Although undesirable behavior following demands likely serves as an escape function, it is possible that the way in which the demand is presented (i.e., how “rude” it is perceived to be) acts as a motivating operation. Therefore, it is important to determine whether it is the demand itself or the delivery (or both) of the demand that is occasioning behavior.

Williams et al. (2008) found that some phrasing of staff demands (e.g., in “elderspeak”) can result in undesired behaviors. Additionally, there is existing evidence that social communications that could be perceived as unacceptable not only might result in undesirable behavior but also may adversely affect someone’s quality of life by affecting relationships between staff and clients. Ryan et al. (1995) showed that communication that is inappropriate for the age and ability of the receiving adult can cause declined health, increased dependent behaviors, and reduced opportunities to interact in conversations. There is a need for training care staff with regard to how to interact with older adults for whom they provide care.

Opportunities for verbal interactions between older adults can decline with age and may be associated with deficits in remembering behavior or separation from family and friends (Robinson et al., 2006). Therefore, a further avenue for enhancing relationships between staff and clients might be to explore ways to facilitate social interactions so that vocal-verbal exchanges are more than just demands. For adults with a learning history of responding to behavior they perceive as unacceptable with rude behavior of their own, it is important to help staff find ways to interact with older adults that result in more pleasant social interactions. We found that a simple demand assessment akin to those conducted by Roscoe et al. (2009) was successful in identifying specific antecedents that occasioned rude responses from our participant. It is possible that what is perceived as unacceptable will vary across older adults (and is likely affected by factors such as varying cultural social rules). Therefore, the development of simple, quick-to-administer assessments like the one we used might help staff tailor the way they interact with the people they support. Care home staff often report that custodial tasks preclude social interactions with residents, and therefore, when a demand is to be presented, it is usually unavoidable (Häggström, Skovdahl, Fläckman, Kihlgren, & Kihlgren, 2004).

We acknowledge that our intervention was composed of two components: phrasing the demand as a mand for assistance and the inclusion of a choice. Although choice as an antecedent has been shown to be effective in increasing compliance with task demands (e.g., Tasky, Rudrud, Schulze, & Rapp, 2008), we were unable to determine whether the offer of a choice or the mand for assistance (or both) was needed for the intervention to be effective. A component analysis would have been an effective way to determine whether one of the two components would have been adequate; however, due to the considered risks and the need for quick intervention (i.e., behaviors occurring in shared environments affecting other residents), one was not conducted.

Although the principles of behavior remain the same, many of our methods, assessments, and interventions may require adaptations for older adults. One unique characteristic of working with older adults with dementia is the complex and long learning histories that may differ from other populations with whom we work (e.g., children with autism spectrum disorder). Some of the resulting difficulties are alluded to in the literature (e.g., confusion during baseline assessment; Raetz, LeBlanc, Baker, & Hilton, 2013), but these have yet to be explored systematically. We suggest that more research is required to explore adaptations of behavioral approaches for adults with dementia and complex or intact vocal-verbal repertoires. For example, more research is required to identify acceptable prompting methods (e.g., during personal care, during which undesirable behavior might occur). Similarly, although stimulus preference assessments have been used with adults with dementia (LeBlanc, Raetz, Baker, Strobel, & Feeney, 2008), there is a paucity of research on how to implement a preference assessment with clients for whom being offered a choice in a highly structured way might seem unusual in the setting. Adaptations may also be required when a person’s intact vocal-verbal repertoire might interfere with methods more commonly used with people with less complex verbal behavior (e.g., experimental functional analyses). We suggest that to facilitate the growth of behavior analysis in older adult settings (i.e., to ensure acceptability and effectiveness), research on how best to adapt our current methods should be a priority for researchers. Our study is a first attempt to assess appropriate adaptations to how demands should be placed that both evoke the behavior required and are socially acceptable to our clients.

## Data Availability

The data that support the findings of this study are available from the corresponding author upon reasonable request.
